# Effects of Calcium Fructoborate on Levels of C-Reactive Protein, Total Cholesterol, Low-Density Lipoprotein, Triglycerides, IL-1β, IL-6, and MCP-1: a Double-blind, Placebo-controlled Clinical Study

**DOI:** 10.1007/s12011-014-0155-9

**Published:** 2014-11-30

**Authors:** Otilia-Constantina Rogoveanu, George Dan Mogoşanu, Cornelia Bejenaru, Ludovic Everard Bejenaru, Octavian Croitoru, Johny Neamţu, Zbigniew Pietrzkowski, Tania Reyes-Izquierdo, Andrei Biţă, Iulia Daria Scorei, Romulus Ion Scorei

**Affiliations:** 1 Department of Physical Medicine and Rehabilitation, University of Medicine and Pharmacy of Craiova, Craiova, Romania; 2 Department of Pharmacognosy and Phytotherapy, University of Medicine and Pharmacy of Craiova, Craiova, Romania; 3 Department of Vegetal and Animal Biology, University of Medicine and Pharmacy of Craiova, Craiova, Romania; 4 Department of Drug Control, University of Medicine and Pharmacy of Craiova, Craiova, Romania; 5 Department of Physics, University of Medicine and Pharmacy of Craiova, Craiova, Romania; 6 Applied BioClinical Laboratory, Futureceuticals, Inc., 16259 Laguna Canyon Rd, Irvine, CA 92618 USA; 7 BioBoron Research Institute, Craiova, Romania

**Keywords:** Clinical trial, Calcium fructoborate, MCP-1, IL-6, IL-1β, Blood lipids, Triglycerides

## Abstract

Calcium fructoborate (CFB) has been reported as supporting healthy inflammatory response. In this study, we assess the effects of CFB on blood parameters and proinflammatory cytokines in healthy subjects. This was a randomized, double-blinded, placebo-controlled trial. Participants received placebo or CFB at a dose of 112 mg/day (CFB-1) or 56 mg/day (CFB-2) for 30 days. Glucose, total cholesterol (TC), low-density lipoprotein (LDL), high-density lipoprotein (HDL), triglycerides (TG), C-reactive protein (CRP), homocysteine, interleukin 1 beta (IL-1β), IL-6, and monocyte chemoattractant protein-1 (MCP-1) were determined before and after supplementation. CFB-1 showed a reduction in blood levels of CRP by 31.3 % compared to baseline. CFB-1 and CFB-2 reduced LDL levels by 9.8 and 9.4 %, respectively. CFB-1 decreased blood homocysteine by 5.5 % compared with baseline, whereas CFB-2 did not have a significant effect. Blood levels of TG were reduced by 9.1 and 8.8 % for CFB-1 and CFB-2, respectively. Use of both CFB-1 and CFB-2 resulted in significantly reduced IL-6 levels, when compared within and between groups. IL-1β was reduced by 29.2 % in the CFB-1 group. Finally, CFB-1 and CFB-2 reduced MCP-1 by 31 and 26 %, respectively. Our data indicate that 30-day supplementation with 112 mg/day CFB (CFB-1) resulted in a significant reduction of LDL, TG, TC, IL-1β, IL-6, MCP-1, and CRP. HDL levels were increased, when compared to baseline and placebo. These results suggest that CFB might provide beneficial support to healthy cardiovascular systems by positively affecting these blood markers (ClinicalTrials.gov, ISRCTN90543844; May 24, 2012 (http://www.controlled-trials.com/ISRCTN90543844)).

## Introduction

Calcium fructoborate (CFB) is a nature-identical mimetic of a molecule naturally present in fruits and is commercially produced by a patented process that was first described by Miljkovic et al. (US Patent #5,962,049) [1]. CFB has previously been reported as supporting healthy inflammatory response [2–4]. More recently published clinical research has shown that CFB may reduce knee discomfort and improve flexibility as measured by subjective evaluations such as the Western Ontario and McMaster Universities arthritis index (WOMAC) score and McGill index [5] and may modulate markers associated with inflammation [4, 6, 7]. In particular, CFB reduces circulating levels of C-reactive protein (CRP) [3, 4], an immune recognition protein that is a sensitive marker of inflammation [4, 8–10].

In general, current scientific evidence supports the hypothesis that the cardiovascular health is directly related and impacted by the body’s inflammatory processes [11]. If this is the case, cardiovascular status could be monitored by measuring certain inflammatory biomarkers [12, 13]. Concomitant evaluation of lipid levels, homocysteine, and CRP has been suggested to predict the risk of cardiovascular [14] and coronary heart [15–17] conditions. Other, more general, markers of inflammation such as interleukin (IL)-1β and IL-6 also provide useful information about cardiovascular risk [18]. Likewise, IL-1β is increasingly becoming recognized as a proatherogenic factor and biomarker of cardiovascular inflammation [19]. The objective of this study was to investigate whether CFB alters blood levels of lipids, homocysteine, CRP, IL-1β, IL-6, and MCP-1 in generally healthy middle-aged subjects in order to evaluate the potential use of CFB as a dietary supplement to support cardiovascular health.

## Subjects and Methods

### Ethics Approval

This trial was approved by the Institutional Ethics Committee of the University of Medicine and Pharmacy of Craiova, Romania, on May 3, 2012. In addition, the trial was performed in accordance with the guidelines put forth in the Declaration of Helsinki of 1975, which was last reviewed in 2008 [20]. An informed consent form was signed by every participant prior to beginning the trial.

### Inclusion and Exclusion Criteria

Male and female participants were considered for inclusion, if they were between the ages of 40 and 60 years and had a body mass index (BMI) between 24 and 27 kg/m^2^, normal blood pressure or minor hypertension (<140/80–90 mmHg), blood CRP >3 mg/L, LDL >130 mg/dL, elevated triglycerides (>200 mg/dL), HDL <40 mg/dL, and fasting glucose <100 mg/dL. Participants were excluded if they had a diagnosis of or symptoms consistent with cardiovascular disorders (e.g., angina, shortness of breath), diabetes mellitus, renal failure, infectious or inflammatory disorders, active allergies, tobacco use, consumption of more than two alcoholic beverages per day, use of any supplements within 30 days prior to the initiation of the study, or use of statins, anti-hypertensive, anti-hyperlipidemia, anti-inflammatory, or anti-diabetic medications.

### Study Design and Intervention Administration

Participants were randomly distributed into three groups with an identical number of females and males per group. Two participants from group A (initial *N* = 28) and one participant from group C (initial *N* = 27) were excluded because of noncompliance. The final number of participants analyzed in each group was 26. Baseline demographic data of the study groups is provided in Table 1. All included subjects were clinically healthy, and there were no significant demographic differences between groups. Subjects did not receive or take any additional nutritional supplements or other related products during the study. Participants were instructed to fast for at least 12 h prior to the beginning of the trial. Supplements were distributed to the participants after initial blood collection on the first day of the trial. All preparations were provided as identically colored capsules in identical bottles. The test formulation was CFB, a patented commercially available dietary supplement that has been tested in three previous clinical studies [4–6]. Group A (CFB-1) received 112 mg/day of CFB given as a 56-mg dose twice daily, which is the equivalent to 3.0 mg boron/day. Group C (CFB-2) received 56 mg/day CFB as a 28-mg dose twice daily, the equivalent to 1.5 mg boron/day. Even though the diet was not monitored, it has been reported that the foods commonly consumed by Romanians in urban and rural zones are within 2.0 ± 0.7 mg/day per person [8]. Group B, the placebo arm, received 80 mg/day fructose given as a 40-mg dose twice daily; this dosage is equal to the amount of fructose present in 112 mg CFB. To ensure compliance, extra capsules were added to each bottle, and periodic phone calls and reminders to the participants were made.

**Table 1:** Baseline demographic characteristics of subjects that successfully completed the study

	Placebo (group B)	CFB-2 (group C)	CFB-1 (group A)
Number of participants per group (*N*)	26	26	26
Men/women (*n*)	10:16	11:15	13:13
Age (mean ± SD) [years]	54 ± 3.2	56 ± 6.8	51 ± 5.6
Environment (rural/urban)	0:26	0:26	0:26
BMI (mean ± SD) [kg/m^2^]	26.34 ± 4.23	25.32 ± 1.69	26.89 ± 4.99

*BMI* body mass index, *CFB* calcium fructoborate, *SD* standard deviation

### Biochemical and Immunological Assessment

For biochemical analyses, samples of fasting venous blood were drawn in the morning of the inclusion day and after 30 days. Blood was drawn from the antecubital vein into “dry” serum tubes (BD Vacutainer, Franklin Lakes, NJ, USA). Upon clotting, blood was centrifuged and serum was collected for analysis of blood chemistry parameters, such as TC, HDL, LDL, TG, glucose, and CRP, using standard biochemical procedures.

### Statistical Analyses

Descriptive statistics are reported as mean ± standard deviation (SD) or median (minimum–maximum) in Table 2. *p* values for each parameter based on comparison of the results at day 1 versus day 30 were calculated using Wilcoxon signed-rank test. Normalized data were calculated based on the percent change from baseline [(measurement at day 30/measurement at day 1) × 100]. We tested the significance of differences in normalized data between groups using the Kruskal–Wallis one-way ANOVA on ranks test. All multiple pairwise comparisons were made using Tukey’s honest significant difference (HSD) post hoc tests for each parameter. In addition, *p* values from Kruskal–Wallis one-way ANOVA test for each parameter were calculated. Percentage change among the groups was analyzed using one-way ANOVA on ranks tests. Finally, *p* values of Tukey’s HSD tests for each comparison between groups were also calculated (Table 3).

**Table 2:** Comparison of blood parameters between day 1 and day 30

	Treatment	Number	Day 1	Day 30	*p* value within-treatment^a^	
			197.6 ± 33.8	186.7 ± 26.7	<0.001	***
Total cholesterol	A	26	189.9 (150.3–267.7)	185.2 (147.1–254.9)		
			194.5 ± 23.2	193 ± 20.4	0.178	
	B	26	191.1 (160.7–254.9)	191.9 (157–243.8)		
			193.4 ± 43.1	180.1 ± 38.1	<0.001	***
	C	26	192.8 (77.4–317.1)	180.9 (73.9–269.2)		
			46.1 ± 11.1	47.7 ± 10.3	0.243	
HDL-Chol.	A	26	45.1 (21.3–63.8)	48.2 (19.3–63.0)		
			45.9 ± 9.6	46.0 ± 9.3	0.611	
	B	26	43.2 (34.2–65.2)	45.2 (30.9–65.0)		
			43.9 ± 8.2	45.7 ± 7.6	0.003	**
	C	26	42.8 (33.1–61.2)	45 (33.9–61.7)		
			145 ± 23.2	129.7 ± 16.4	<0.001	***
LDL-Chol.	A	26	148.7 (103.0–188.7)	128.0 (98.3–160.5)		
			132.0 ± 19.7	129.6 ± 18.4	0.0912	
	B	26	134.6 (100.1–185.4)	128.9 (103.8–183.9)		
			139.3 ± 24.8	125.8 ± 24.1	<0.001	***
	C	26	138.7 (96.9–202.0)	122.8 (74.8–169.3)		
			163.2 ± 39.4	145.5 ± 25.4	<0.001	***
Triglycerides	A	26	155.9 (117.0–254.6)	140.0 (104.6–211.1)		
			165.1 ± 33.0	161.5 ± 26.0	0.075	
	B	26	163.9 (120.0–246.6)	161.3 (121.4–214.3)		
			181.4 ± 33.1	163.9 ± 23.4	<0.001	***
	C	26	185.0 (122.3–238.1)	164.9 (115.6–200.2)		
			78.7 ± 12.0	76.3 ± 11.9	0.007	**
Glucose	A	26	81.6 (60.6–100.1)	75.3 (58.7–98.5)		
			73.7 ± 8.6	73.1 ± 7.8	0.424	
	B	26	71.0 (61.2–99.3)	73.1 (63.2–99.1)		
			75.0 ± 11.3	75.3 ± 10.9	0.859	
	C	26	71.7 (54.2–100.2)	76.1 (54.0–97.9)		
			3.6 ± 3.0	2.4 ± 2.0	<0.001	***
hs-CRP	A	26	2.8 (0.6–12.4)	1.6 (0.5–8.2)		
			3.5 ± 2.9	3.0 ± 1.9	0.086	
	B	26	2.9 (0.6–11.3)	2.9 (0.1–7.7)		
			3.6 ± 2.2	2.7 ± 1.4	0.031	*
	C	26	3.6 (0.2–7.6)	2.5 (0.4–5.7)		
			16.8 ± 10.4	14.4 ± 8.9	0.004	**
Homocysteine	A	26	14.5 (2.5–41.2)	12.6 (2.3–37.4)		
			19.6 ± 12.7	19.1 ± 12.6	0.374	
	B	26	17.2 (5.4–55.2)	11.7 (6.8–53.8)		
			16.4 ± 9.9	15.0 ± 9.1	0.091	
	C	26	13.4 (5.2–38.2)	11.3 (4.7–35.6)		
			3.2 ± 2.6	2.0 ± 1.8	<0.001	***
IL-1β	A	26	2.5 (0.4–9.4)	1.2 (0.4–6.3)		
			2.2 ± 1.8	2.1 ± 1.8	<0.001	***
	B	26	1.7 (0.5–7.8)	1.4 (0.3–7.4)		
			3.7 ± 3.0	2.8 ± 2.4	<0.001	***
	C	26	3.1 (0.5–12.4)	2.3 (0.3–11.7)		
			8.2 ± 9.3	6.7 ± 7.8	<0.001	***
IL-6	A	26	6.4 (0.4–44.2)	5.4 (0.4–35.7)		
			8.8 ± 8.4	8.2 ± 7.8	<0.001	***
	B	26	5.3 (0.8–32.3)	5.1 (0.6–29.7)		
			7.8 ± 6.7	6.7 ± 7.8	<0.001	***
	C	26	6.3 (0.3–20.9)	5.3 (0.2–20.7)		
			281.7 ± 53.5	195.3 ± 42.9	<0.001	***
MCP-1	A	26	277.4 (184.6–380.4)	191.4 (110.4–270.1)		
			305.1 ± 81.7	256.0 ± 63.8	<0.001	***
	B	26	273.4 (215.5–507.4)	243.5 (134.6–383.7)		
			291.8 ± 68.7	221.8 ± 36.9	<0.001	***
	C	26	283.5 (181.3–483.6)	212.6 (169.1–290.4)		

Mean values (±SD) and median values (range) for each parameter and time point are provided (*N* = 26 within each study group). *p* values were obtained by Wilcoxon signed-rank tests. Group A, CFB-1 (112 mg/day CFB); group B, placebo; group C, CFB-2 (56 mg/day CFB)

^a^ *p* values are from Wilcoxon signed-rank test

***p < 0.001; **p < 0.01; *p < 0.05

**Table 3:** Summary of changes from baseline between groups and pairwise multiple comparisons for each parameter

	Group A	Group B	Group C	*p* value between-groups^a^		Post hoc comparison	Adjusted *p* value^b^	
Total cholesterol	95.1 ± 5.8	99.5 ± 5.7	93.4 ± 5.1	<0.001	***	B-A	0.027	*
	95.4 (84.6–106.9)	99.2 (82.0–15.3)	95.2 (82.6–101.8)			C-A	0.667	
						C-B	<0.001	***
HDL-Chol.	105.8 ± 20.9	100.5 ± 8.6	104.6 ± 6.7	0.207				
	103.9 (62.1–171.8)	101.0 (76.5–18.7)	104.7 (89.0–118.5)					
LDL-Chol.	90.2 ± 7.7	98.6 ± 7.1	90.6 ± 10.3	<0.001	***	B-A	<0.001	***
	91.4 (75.3–101.0)	99.2 (76.4–111.3)	96.4 (70.0–102.7)			C-A	0.816	
						C-B	0.01	**
Triglycerides	90.9 ± 10.4	99.0 ± 11.1	91.2 ± 7.1	<0.001	***	B-A	0.007	**
	91.5 (72.4–113.3)	98.8 (73.0–129.3)	93.4 (78.7–99.7)			C-A	0.965	
						C-B	0.003	**
Glucose	97.1 ± 6.7	99.4 ± 3.8	100.5 ± 4.0	0.048	*	B-A	0.27	
	98.3 (81.6–111.0)	98.5 (90.4–105.4)	99.5 (94.4–111.0)			C-A	0.046	*
						C-B	0.813	
hs-CRP	68.7 ± 14.4	89.5 ± 36.3	118.1 ± 139.9	0.045	*	B-A	0.022	*
	70.6 (41.3–92.3)	86.4 (16.7–209.1	77.7 (30.8–700.0)			C-A	0.569	
						C-B	0.803	
Homocysteine	94.5 ± 54.1	101.9 ± 30.2	97.7 ± 32.3	0.119				
	81.1 (55.1–334.1)	96.2 (43.0–173.9)	87.9 (52.0–177.1)					
IL-1β	70.8 ± 32.3	84.1 ± 15.0	78.0 ± 15.3	0.022	*	B-A	0.035	*
	75.0 (16.7–171.4)	88.2 (55.6)–104.7)	82.3 (35.7–100.0)			C-A	0.242	
						C-B	0.305	
IL-6	80.8 ± 12.5	90.8 ± 10.4	79.6 ± 16.3	0.008	**	B-A	0.025	*
	79.5 (42.9–100.0)	93.6 (66.7–109.5)	82.3 (35.7–100.0)			C-A	1	
						C-B	0.032	*
MCP-1	70.1 ± 12.7	87.2 ± 20.2	78.7 ± 15.7	<0.001	***	B-A	0.002	**
	70.7 (43.7–99.6)	96.3 (28.2–107.9)	81.6 (46.7–107.1)			C-A	0.078	
						C-B	0.033	*

Changes from baseline (% change) for each parameter are summarized as mean ± SD or median (range). Group A, CFB-1 (112 mg/day CFB); group B, placebo; group C, CFB-2 (56 mg/day CFB)

^a^ *p* values are from Kruskal-Wallis one-way ANOVA test

^b^Adjusted *p* values are from Tukey’s HSD test

***p < 0.001; **p < 0.01; *p < 0.05

### Safety Assessment

Tolerance was evaluated at each visit by asking subjects about the appearance of any unexpected side effects.

## Results

### Participant Demographics

**Fig. 1:**
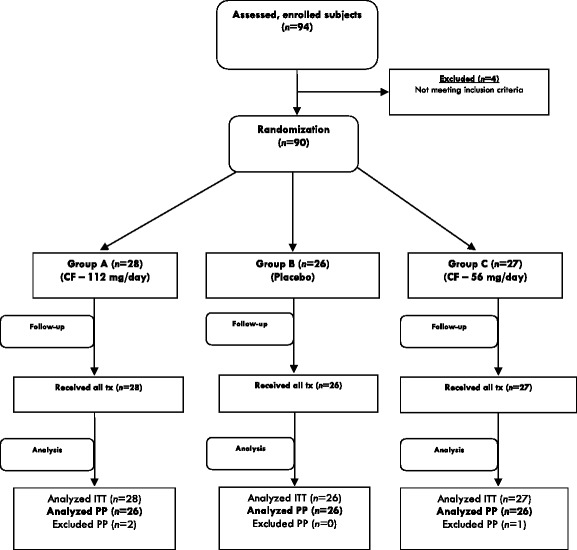
Flowchart for subject recruitment and study progression. *CF* calcium fructoborate, *ITT* intention to treat, *PP* patients, *tx* study treatment

The flow chart for subject recruitment and the various steps of this double-blinded, placebo-controlled study is presented in Fig. 1. The total number of enrolled subjects was 94. Of these, four individuals were found to have diabetes and were excluded from the trial, and 90 met the inclusion criteria. These subjects were randomized into three groups: placebo, CFB at a dose of 112 mg/day (CFB-1), and CFB at a dose of 56 mg/day (CFB-2). Twelve subjects were later excluded because of lack of compliance; therefore, 78 subjects completed the entire protocol, and the final number of subjects analyzed in each group was 26. After these adjustments, the final study population included a higher number of females than males (56 and 44 %, respectively).

### Analysis Within Study Groups (Day 1 Versus Day 30)

No adverse effects were reported for either of the supplementation doses. Table 2 presents statistical analyses of the lipid profile, fasting glucose, homocysteine, and CRP values for all groups of the study population. A significant decrease in the levels of TC, LDL, TG, and CRP was observed in groups A and C but not in group B. The levels of glucose and homocysteine were significantly decreased in the CFB-1 supplementation group but were not significantly altered in the placebo or CFB-2 supplementation groups. Interestingly, HDL was significantly increased by CFB-2, but not by CFB-1 or placebo. The Pearson’s correlation coefficients for group A (CFB-1) were 0.96 for TC (*p* < 0.001), 0.85 for LDL (*p* < 0.001), 0.90 for TG (*p* < 0.001), 0.94 for CRP (*p* < 0.001), 0.82 for homocysteine (*p* = 0.004), and 0.91 for glucose (*p* < 0.007). For group C (CFB-2), the Pearson’s correlation coefficients were 0.94 for TC (*p* < 0.001), 0.95 for HDL (*p* < 0.003), 0.83 for LDL (*p* < 0.001), 0.91 for TG (*p* < 0.001), and 0.54 for CRP (*p* < 0.031).

### Analysis Between Study Groups

Table 3 shows analysis based on change from baseline serum levels over 30 days. Significant differences were found between groups A (CFB-1) and B (placebo), and between groups C (CFB-2) and B (placebo) for TC, LDL, and TG, which showed significantly lower levels in both test groups (CFB at 112 or 56 mg) compared with the placebo group. HDL levels were significantly higher in the CFB-2 group compared with the placebo control. CRP levels were significantly lower in the CFB-1 group compared to placebo. Use of CFB-1 resulted in a significant decrease in IL-1β levels compared to placebo. Similarly, the CFB-2 group showed a slight decrease in IL-1β, but this value was not significantly different from that in the placebo group. Study subjects using CFB-1 and CFB-2 (groups A and C) experienced a statistically significant reduction in the level of IL-6 and MCP-1 compared to the placebo group.

## Discussion

Lipids and lipoproteins are well-known risk factors for development of heart conditions [14]. Elevated levels of TG, TC, and LDL, as well as low levels of HDL, are well-documented risk factors for atherogenesis [21]. In addition, CRP and homocysteine have emerged as important indicators of risk for cardiovascular conditions [14, 15]. Although they are not currently routinely included in clinical cardiovascular risk assessments, various proinflammatory biomarkers have been strongly associated with the development of cardiovascular conditions. One of the most intriguing of these is MCP-1. MCP-1 is produced by various cell types within the arterial wall, and its expression can be induced by a number of substances including cytokines [22], minimally modified LDL [23], homocysteine [24], shear stress [25], among other factors [26]. Expression of MCP-1 by macrophages increases the progression of atherosclerosis [27, 28]. Conversely, it has been reported that MCP-1 knockout mice are markedly protected from macrophage recruitment and atherosclerotic lesions [29]. In humans, plasma levels of MCP-1 correlate with the severity of cardiovascular conditions [30, 31]. Several researchers have postulated that blocking or reducing the expression of MCP-1 might be beneficial in preventing the development of unhealthy heart conditions [26, 32].

IL-1β and IL-6 are cytokines that provide clues to the presence of inflammatory processes. Systemic increases in these inflammatory molecules, together with tumor necrosis factor and CRP, occur during the atherosclerosis process [33, 34]. IL-1β modulates the atherogenic process by contributing to the arterial wall inflammation, leukocyte chemotaxis/adhesion, and the rupture of atherosclerotic plaques [35]. Therefore, blood levels of IL-6 alone or in combination with other biomarkers are predictive of coronary pathologies [18, 36, 37]. It therefore follows that a reduction in the blood levels of these inflammatory cytokines could be considered as an approach to support healthy cardiovascular systems and cardiovascular health in healthy subjects [38].

Our results suggest that use of CFB at a daily dose as low as 112 mg for 30 days may significantly reduce levels of the proinflammatory and proatherogenic markers TC, LDL, triglycerides, CRP, and homocysteine while increasing the levels of HDL, which is considered a protective lipid. Furthermore, supplemental use of CFB at a dose of 112 mg per day appears to have a statistically significant inhibitory effect on proinflammatory cytokines such as IL-1β, IL-6, and MCP-1.

### Limitation

Further studies with larger groups of subjects are necessary to verify and confirm the effects of CFB on proinflammatory markers. Also, a study with a longer timeframe could establish the long-term benefits of CFB supplementation.

## Conclusions

Under these experimental conditions, we observed a statistically significant reduction in the blood levels of TC, LDL, TG, CRP, homocysteine, IL-1β, IL-6, and MCP-1 and an increase in the level of HDL. Consequently, CFB exerts beneficial effects on these subclinical blood markers within a healthy population. This study highlights the ability of CFB to maintain a healthy lipid profile (especially for LDL and TG) and to support the maintenance of healthy blood levels of proinflammatory markers in middle-aged healthy subjects.

## Abbreviations

BMD: Bone mineral density
BMI: Body mass index
CFB: Calcium fructoborate
CFB-1: 112 mg of CFB per dose
CFB-2: 56 mg of CFB per dose
Chol.: Cholesterol
CRP: C-reactive protein
HDL: High-density lipoprotein
IL-1β: Interleukin 1 beta
IL-6: Interleukin 6
LDL: Low-density lipoprotein
MCP-1: Monocyte chemoattractant protein-1
SD: Standard deviation
TC: Total cholesterol
TG: Triglycerides
